# [Retracted] Propofol modulates the proliferation, invasion and migration of bladder cancer cells through the miR‑145‑5p/TOP2A axis

**DOI:** 10.3892/mmr.2024.13218

**Published:** 2024-04-08

**Authors:** Yi Du, Xudong Zhang, Hongwei Zhang, Yiding Chen, Shuying Zhu, Jinjun Shu, Hui Pan

Mol Med Rep 23: 439, 2021; DOI: 10.3892/mmr.2021.12078

Following the publication of this paper, it was drawn to the Editor's attention by a concerned reader that the tumour images shown in Fig. 6B on p. 8 were strikingly similar to data appearing in different form in other articles written by different authors at different research institutes, which had either already been published or were under consideration for publication at around the same time. Owing to the fact that the contentious data in the above article were already under consideration for publication prior to its submission to *Molecular Medicine Reports*, the Editor has decided that this paper should be retracted from the Journal. The authors were asked for an explanation to account for these concerns, but the Editorial Office did not receive a reply. The Editor apologizes to the readership for any inconvenience caused.

